# Diabetic ketoacidosis and coronavirus disease 2019-associated mucormycosis: a case report

**DOI:** 10.1186/s13256-022-03594-2

**Published:** 2022-10-31

**Authors:** Vanessa Monroig, Keiko M. Tarquinio

**Affiliations:** 1 Department of Pediatrics, Emory University School of Medicine, Atlanta, GA USA; 2 Division of Pediatric Critical Care Medicine, Children’s Healthcare of Atlanta, Atlanta, GA USA

**Keywords:** Mucormycosis, Diabetes, Diabetic ketoacidosis, COVID-19, Case report

## Abstract

**Background:**

Mucormycosis is a rare, life-threatening fungal infection that affects immunocompromised hosts. Diabetes mellitus is a common predisposing condition and most often presents with rhino-orbital-cerebral infection. Association with coronavirus disease 2019 infection was revealed following a resurgence in cases of mucormycosis during the second wave of the pandemic wherein poorly controlled diabetes mellitus was the most significant risk factor in the affected population. Rhino-orbital-cerebral mucormycosis has a high mortality rate, and cerebral involvement is a poor prognostic factor. Herein, we report a case of newly diagnosed diabetes mellitus with concurrent coronavirus disease 2019 infection complicated by diabetic ketoacidosis and rhinocerebral mucormycosis at presentation, describe the diagnostic and therapeutic challenges, and discuss the interventions that ultimately resulted in a favorable clinical response.

**Case presentation:**

We describe the case of a previously healthy 13-year-old African American female patient with newly diagnosed diabetes mellitus and concurrent severe acute respiratory syndrome coronavirus 2 infection whose disease course was complicated by rhinocerebral mucormycosis. She presented with fever, altered mental status, and Kussmaul respirations and was diagnosed with diabetic ketoacidosis with concern for cerebral edema. Concern for infectious cerebritis arose due to recurring fevers and persistently altered mental status despite correction of her metabolic derangements. This raised concern for infectious cerebritis and prompted evaluation with serial head imaging, lumbar puncture, and initiation of broad empiric antimicrobial regimen. Head imaging revealed an evolving cerebral abscess, and fungal deoxyribonucleic acid was identified on blood metagenomics testing, which ultimately confirmed the diagnosis of rhinocerebral mucormycosis. Treatment was challenging as she required surgical debridement of the frontal lobe and aggressive antifungal therapy complicated by electrolyte derangements and electrocardiogram changes that necessitated modification of the antimicrobial regimen. Despite these challenges and high mortality rate, the patient was discharged from the hospital in stable condition to inpatient rehabilitation service for reconditioning after prolonged hospitalization.

**Conclusion:**

Rhinocerebral mucormycosis mortality is associated with delays in therapeutic interventions, thus a high index of suspicion and early recognition were essential for timely initiation of antifungal therapy and surgical debridement.

## Introduction

Mucormycosis is a rare, life-threatening infection caused by fungi that belong to the order *Mucorales* [1, 2]. *Mucorales* are ubiquitous in Nature and are found on decaying vegetables and soil; the genera most implicated in human disease is *Rhizopus *[3]. Mucormycosis has varied clinical presentations that include rhino-orbital-cerebral, pulmonary, cutaneous, and disseminated [4]. The disease is characterized by tissue infarction and necrosis owing to invasion of the host’s vasculature by fungal hyphae [5]. Mucormycosis occurs most often in immune-compromised hosts; risk factors include diabetes mellitus, phagocyte dysfunction (prolonged neutropenia or glucocorticoid use), and iron overload [5, 6]. The coronavirus disease 2019 (COVID-19) pandemic has led to a resurgence in cases of mucormycosis, most notably in India, where 47,000 cases were reported during its second wave of the pandemic (between May and July 2021) [7]. In India, the most significant contributing risk factor was poorly controlled diabetes mellitus, while hematological malignancies and organ transplant recipients were more commonly reported in the rest of the world [7, 8]. There are reports of increased incidence of diabetic ketoacidosis in adults during the COVID-19 pandemic [9, 10]. A retrospective cohort study of adults hospitalized with COVID-19 identified ketosis at presentation in both diabetic and nondiabetic patients. The study concluded that acute COVID-19 infection induces ketosis and, therefore, could precipitate diabetic ketoacidosis in diabetic patients [10]. The mechanism of COVID-19-associated mucormycosis is poorly understood, but proposed mechanisms include poorly controlled hyperglycemia, immune dysregulation (directly or indirectly from COVID-19 infection), and impaired phagocyte function secondary to steroid use [8].

## Case presentation

A previously healthy 13-year-old African American female (body mass index 19.2 kg/m^2^, 55.99% for age and sex percentile) presented to the emergency department with a chief complaint of altered mental status and difficulty breathing. Symptoms were preceded by 1 day of headache and fever, prompting evaluation at an urgent care center where she was found to be SARS-CoV-2 positive. On the morning of presentation, she sought further evaluation in the emergency department for symptom progression to chest pain, heavy breathing, and confusion. Pertinent examination findings at presentation included disorientation, combative behavior without focal neurologic deficit, tachycardia, Kussmaul breathing, dry mucus membranes, and diffuse abdominal tenderness. Laboratory findings revealed leukocytosis (white blood cell count 22.3 × 10^3^/mcL), anion-gap metabolic acidosis (pH 6.92, pCO_2_ < 5 torr, base deficit 28), and hyperglycemia (glucose 668 mg/dL). Urinalysis was significant for ketonuria and glucosuria. Physical examination and laboratory findings were consistent with newly diagnosed diabetes mellitus (hemoglobin A1C > 16%) complicated by diabetic ketoacidosis (DKA), likely triggered by acute COVID-19 infection, with features concerning for cerebral edema. She was started on continuous insulin infusion (0.1 units/kg/h), given a bolus of 3% hypertonic saline (HTS), and was admitted to the pediatric intensive care unit (PICU) for further management. DKA was treated according to the institutional protocol with intravenous fluids, continuous insulin infusion, and close electrolyte monitoring.

**Fig. 1:**
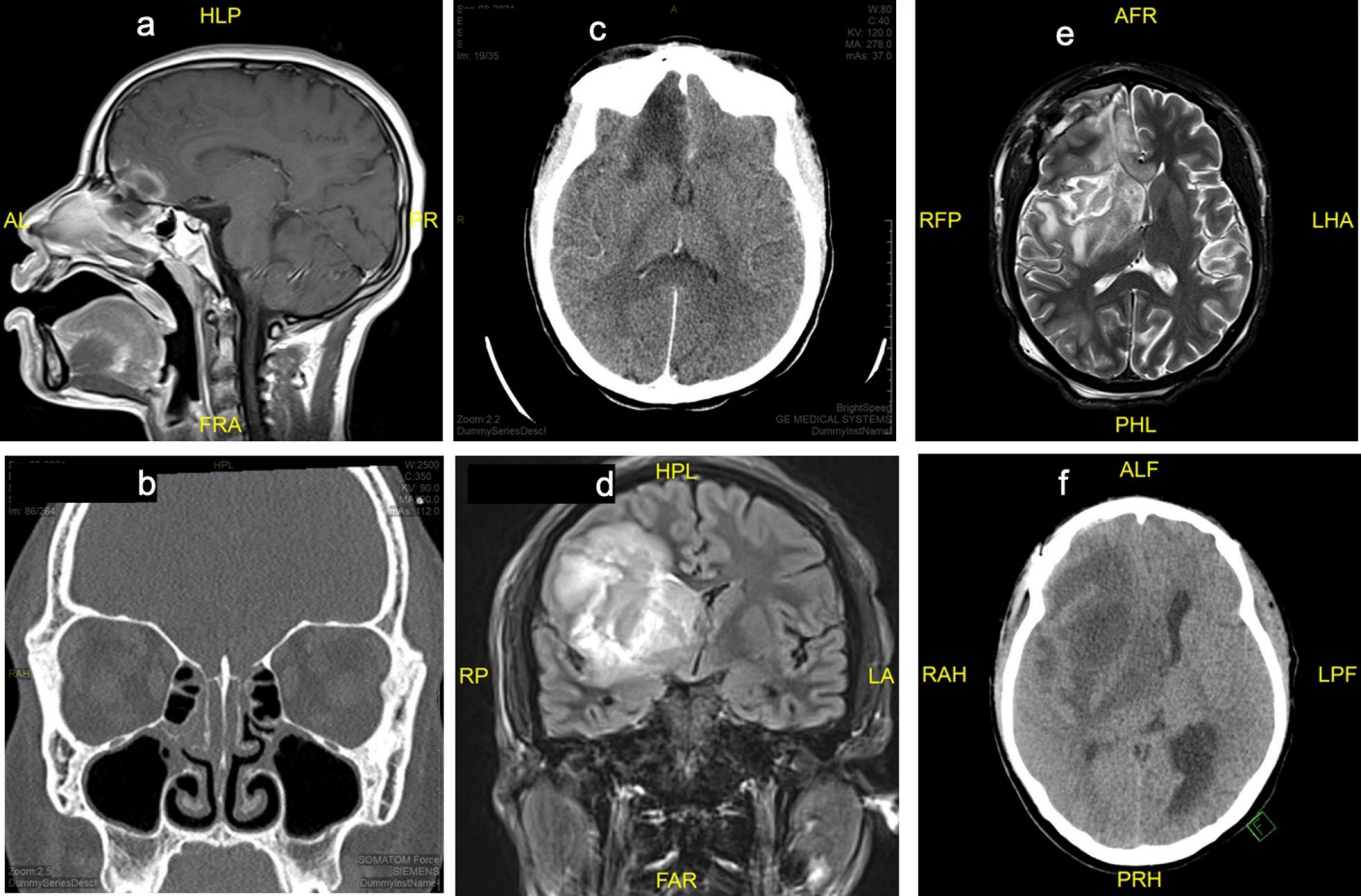
Head images (CT and MRI). **a** (Day 2 admission): MRI T1 brain sagittal view showed abnormal enhancement within the frontal lobe extending from the olfactory floor upward through the right gyrus rectus. **b** (Day 3 admission): sinus CT showed mild opacification of superior maeti, maxillary ostium, medial and posterior ethmoid air cells with erosive changes of the osseous septa and possible defect. **c** (Day 4 admission): head CT showed cerebral edema with mass effect suggestive of infectious cerebritis. **d** (Day 10 admission): MRI Fluid-attenuated inversion recovery (FLAIR) brain coronal view showed significantly increased area of cerebritis/phlegmon involving right frontal lobe extending into the basal ganglia. Interval increased midline shift with extension of vasogenic edema to the left gyrus rectus. **e** (Day 16 admission): MRI T2 brain horizontal view showed progression of parenchymal mucormycosis involving right frontal lobe, right parietal lobe, and across the corpus callosum to the contralateral side with a 3-mm right-to-left midline shift. **f** (Day 25 admission, day 15 post ope, 9/29 images): increased mass effect with right-to-left midline shift with interval increased hypodensity involving the right frontotemporal lobes, basal ganglia, and thalamus extending to the right midbrain, representing increased edema or infection

Due to persistent alteration in mental status shortly after admission, she received two additional HTS boluses and had head computed tomography (CT) scan, which was normal. Despite these interventions and resolution of metabolic derangements, her mentation remained altered. On day 2 of admission, a brain magnetic resonance imaging (MRI) scan (Fig. 1a) showed abnormal enhancement within the frontal lobe extending from the olfactory floor upward. Additional findings were notable for dehiscence of the planum sphenoidale and involvement of the left nasal cavity near superior turbinate, which was concerning for an infectious cerebritis with possible developing phlegmon/intracranial abscess. In the clinical setting of DKA, these findings were concerning for invasive sinusitis with intracranial extension. Lumbar puncture (LP) was subsequently performed on day 2 and revealed cerebrospinal fluid (CSF) pleocytosis with neutrophilic predominance and negative meningitis encephalitis panel. CSF cultures were not obtained at that time due to limited sample volume. An infectious diseases specialist was consulted and recommended an empiric antimicrobial regimen with ceftriaxone, metronidazole, and liposomal amphotericin B given the risk for invasive fungal disease. The patient did not meet criteria for treatment of acute COVID-19 infection, so steroids and antivirals were deferred.

On day 3 of admission, sinus CT scan (Fig. 1b) revealed mild opacification of the superior maeti, maxillary ostium, medial and posterior ethmoid air cells with erosive change of the osseous septa and possible bone defect. An otorhinolaryngology (ENT) surgeon performed an endoscopic nasal examination, notable for friable tissue without evidence of necrosis. Due to limited patient tolerance during the awake examination, biopsies were not obtained at that time. On day 4 of admission, repeat head CT (Fig. 1c) showed interval progression of intracranial pathology with mass effect and cerebral edema suggestive of infectious cerebritis. Repeat LP was unsuccessful, so blood metagenomics testing (Karius test, Redwood City, CA) was obtained on day 5 to aid in the diagnosis. She continued to have daily fevers despite broad-spectrum antimicrobial coverage. Blood cultures obtained at initial presentation remained negative, and an evaluation for endocarditis as a potential source of fever was unrevealing. Invasive fungal disease was most concerning among the differential diagnoses, so posaconazole was added to the antimicrobial regimen on day 7 of admission while awaiting results of the Karius test. Results returned on day 9 of admission and revealed genetic material belonging to the fungal organism *Rhizopus delemar*. The positive Karius test in the setting of progressive intracranial disease confirmed the diagnosis of rhinocerebral mucormycosis.

**Fig. 2:**
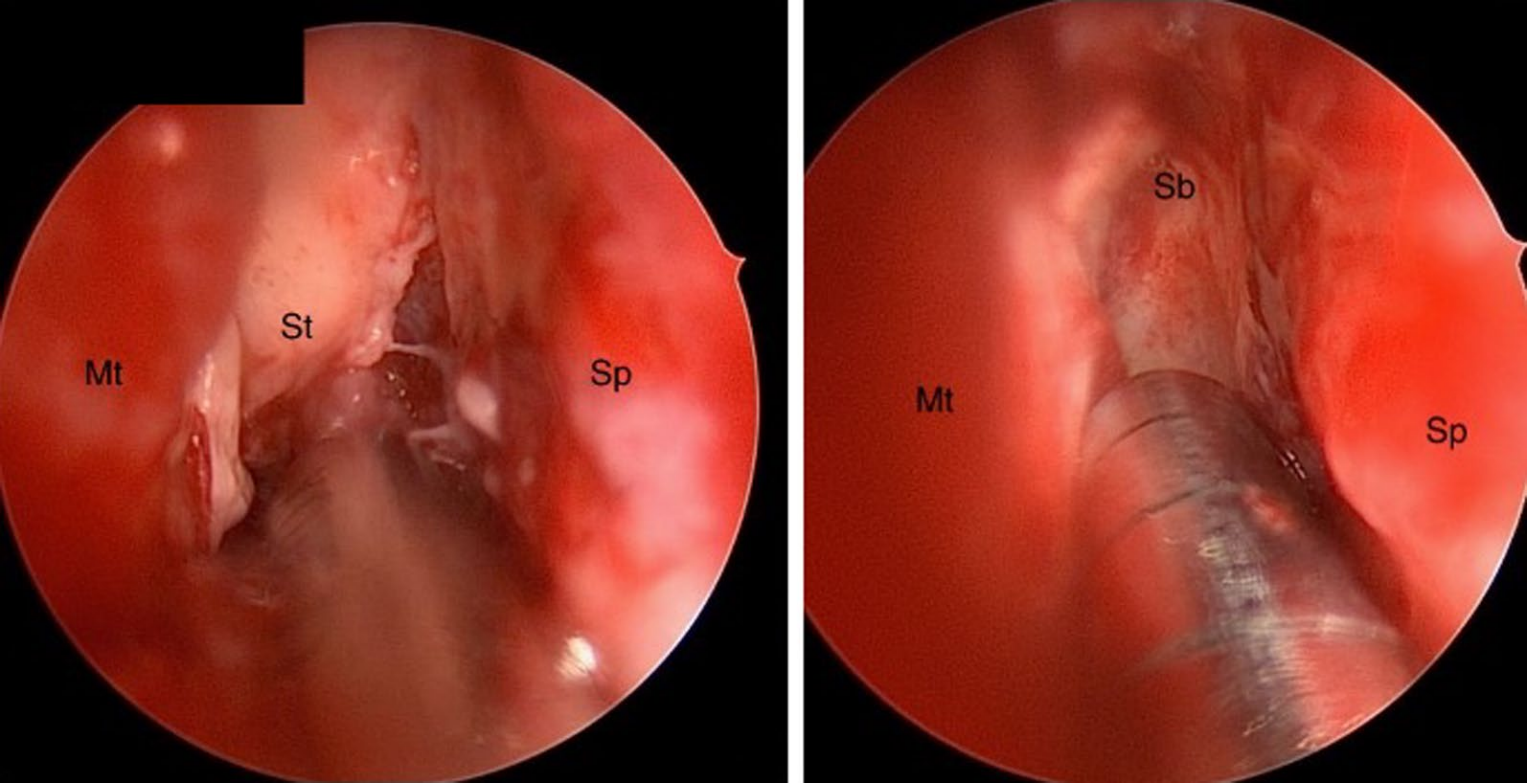
Endoscopic view of the right nasal cavity. Avascular fibrinous conversion of the superior turbinate (St) extends to the level of the anterior skull base (Sb) without classic black necrotic eschar typically visualized with invasive fungal sinusitis. Left picture shows a superior endoscopic view of the right nasal cavity (left picture). Septum—Sp; middle turbinate—Mt; superior turbinate attachment at the anterior skull base—Sb; septum—sp; middle turbinate—Mt

**Fig. 3:**
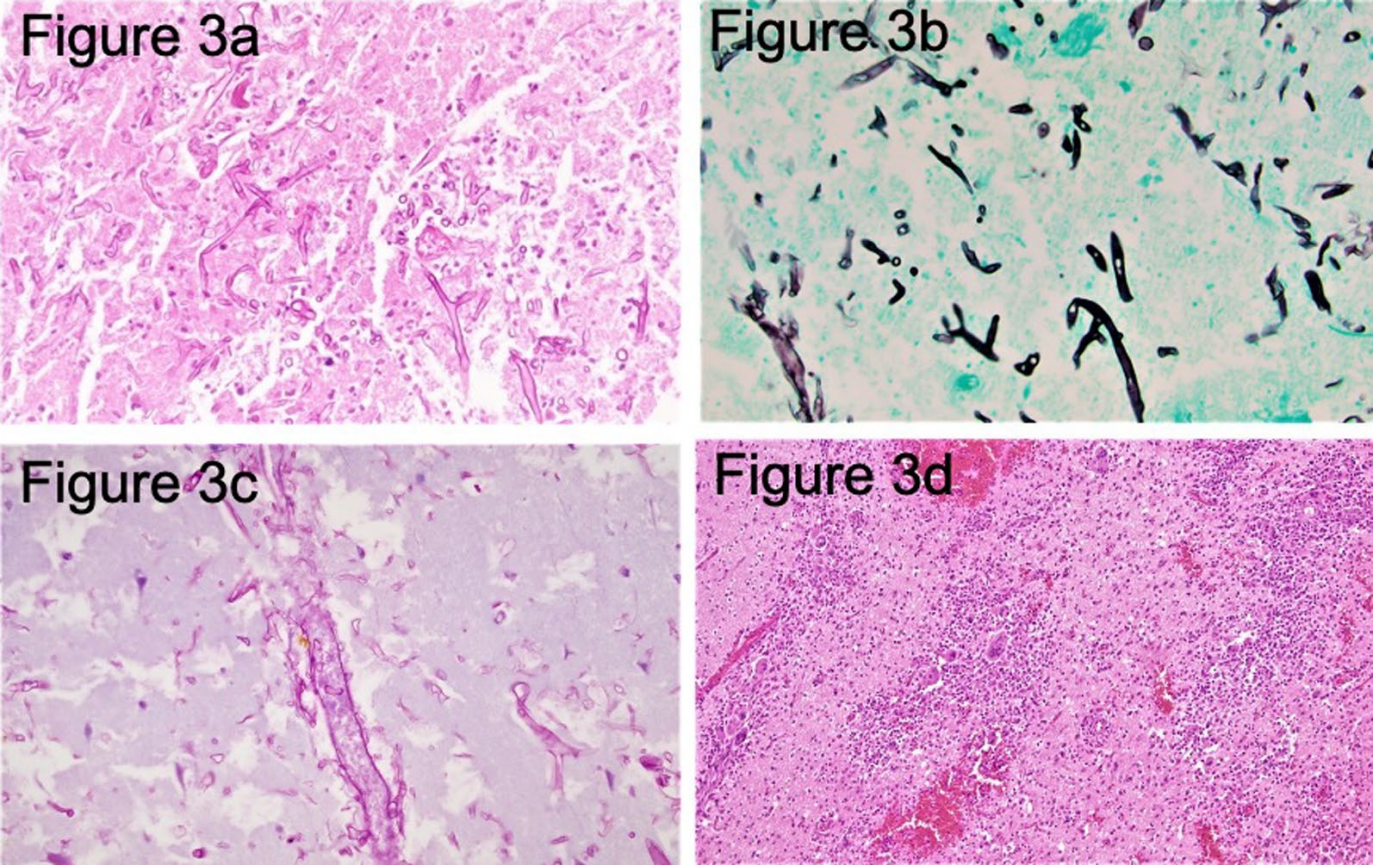
Pathology findings. Microscopic examination of the debridement specimen from right frontal lobe reveals necrotic brain tissue with broad-based ribbon-like pauciseptate hyphae. The angle of branching is wide-angle and greater than other organisms, usually approaching 90° (**a** and **b**). Angioinvasion is easily identified (**c**). There is a marked inflammatory response which is neutrophilic and granulomatous between the viable brain tissue and necrosis (**d**). **a** (Periodic acid–Schiff stain) and **b** (Grocott–Gomori methenamine silver stain): Irregular, broad-based, pauciseptate thick fungal hyphae branching at right angles in the necrotic focus (original magnification, ×400). **c** Angioinvasion or invasion of the vessels by hyphae. **d** (Hematoxylin–eosin stain): Granulomatous inflammatory response (original magnification, ×400)

Vancomycin, ceftriaxone, and metronidazole were discontinued, and treatment with liposomal amphotericin B and posaconazole was continued at that time. Repeat imaging on day 10 (Fig. 1d) showed disease progression with worsening midline shift while on maximal antifungal therapy, prompting the decision to pursue emergent surgical debridement. The patient underwent frontal craniotomy with drainage of fungal abscess and sphenoidotomy with debridement. Intraoperative findings were notable for invasive fungal infection and necrotizing cerebritis (Fig. 2). Pathology revealed necrosis with non-septate broad hyphae and scattered giant cells with micro-abscess formation, and tissue culture isolated *Rhizopus* species (Fig. 3a–d). Posaconazole was discontinued on day 13 due to prolonged QTc interval identified on surveillance electrocardiogram (EKG) and was replaced with isavuconazole. Nasal amphotericin irrigations were performed by ENT as adjunctive therapy.

The patient suffered from central diabetes insipidus and neurological deficits including left-sided hemiparesis and persistent fluctuations in mental status. Weekly head imaging was performed for disease surveillance and showed progression of disease despite maximal antifungal therapy. On postoperative day 6 (day 16 of admission), brain MRI (Fig. 1e) showed extension of intracranial infection to the contralateral hemisphere and brainstem involvement. Given these findings, no further neurosurgical intervention was recommended. Progression of the disease continued, and subsequent imaging (Fig. 1f) showed worsening midline shift (1.5 cm on postoperative day 15) with evidence of early temporal lobe herniation. Hospital course was additionally complicated by amphotericin nephrotoxicity, aspiration pneumonia, and venous catheter-associated thrombus formation requiring anticoagulation therapy. Nasal amphotericin irrigations were discontinued on hospital day 20 due to severe nosebleeds during the procedure.

Supportive care was offered for the aforementioned complications, and the patient continued maintenance therapy with liposomal amphotericin B and isavuconazole. Dose adjustments were made to the antifungals due to renal impairment. Despite these challenges, her clinical status steadily improved. She was transferred to an inpatient rehabilitation facility on hospital day 79 for ongoing medical management and intensive therapies to maximize functional status. After 104 days total of inpatient management, the patient was discharged home and transitioned to outpatient care. Amphotericin B was discontinued at time of discharge, and she remains on isavuconazole monotherapy. As of 10 months from initial diagnosis, she continues showing improvement in functional domains as she can fully ambulate and perform activities of daily living with minimal assistance.

## Discussion

Mucormycosis is an invasive fungal disease caused by organisms of the *Mucorales* order. The most common species implicated in human diseases belong to the genera *Rhizopus *[11]. The *Mucorales* are found ubiquitously in Nature and rarely cause disease in immunocompetent hosts. *Mucorales* can gain entry to a susceptible host through inhalation, ingestion of contaminated food, or abraded skin, which results in the following presentations: rhino-orbital-cerebral, pulmonary, gastrointestinal, cutaneous, and disseminated mucormycosis [4, 12]. Risk factors for disease include diabetes mellitus, hematologic malignancies, hematopoietic stem cell transplant, solid organ transplant, neutropenia, glucocorticoid use, and iron overload [13, 14]. In patients with diabetes mellitus, the most common presentation (with the highest mortality) is rhino-orbital-cerebral mucormycosis [4, 14]. Each form of the disease is characterized by invasion of vascular tissue, resulting in infarction and tissue necrosis [12]. *Mucorales* invades endothelial cells by expressing coat homolog (CotH) proteins that bind to the glucose regulated protein-78 (GRP78) receptor on host cells. Binding stimulates endothelial endocytosis, thus serving as a portal of entry for the fungal organism [11, 15]. Elevated glucose concentrations increase GRP78 expression, which leads to enhanced fungal invasion. *In vitro* studies have demonstrated that ketoacids increase the expression of fungal CotH proteins, highlighting a key predisposing factor for invasive fungal disease in patients with DKA [11].

Olfactory dysfunction has been described in association with novel coronavirus (SARS-CoV-2) infection [16]. This gives rise to the question of whether the virus causes inflammation directly (or indirectly) to the sinus/olfactory nerve? Was this the cause or result of weakening of the osseous septa/ethmoid sinus, ultimately granting the pathogen direct passage to intracranial space? Few studies describe the mechanism of COVID-19 infection resulting in central nervous system diseases. However, viral invasion via the hematogenous route and neural invasion via nerve terminals, such as the olfactory nerve, have been speculated [17]. SARS-CoV-2 can directly interact with angiotensin-converting enzyme 2 (ACE2) receptor via its spike protein to enter the human cell. ACE2 receptors are not only expressed in alveolar epithelial cells [18] but also in brain and glial cells [17]. Thus, the olfactory epithelium (OE) could serve an entry point or reservoir for SARS-CoV-2 replication, which can facilitate SARS-CoV-2 neurotropism [19, 20]. Additionally, poorly controlled diabetes (or newly diagnosed in this patient) may have contributed to immunosuppression, in addition to acute COVID-19 infection, which may have rendered her susceptible to development of rhinocerebral mucormycosis.

Typically, DKA-associated cerebral edema does not result in persistent altered mentation, especially when HTS is administered. New-onset diabetes patients rarely develop rhinocerebral mucormycosis, especially considering previously healthy children in the USA. One study identified only 44 proven pediatric cases from 15 countries over 10 years, and diabetes mellitus accounted for 4.8% with crude mortality of 33.3%, predominantly in patients with malignancy [13]. Failure to respond to routine DKA management prompted further investigation of intracranial pathology and early empiric treatment with antifungal regimen (given persistently high fevers). Adjunctive antifungal therapy was added on presentation while awaiting results of confirmatory testing. Diagnosis was confirmed on day 9 of presentation, and our patient progressed despite maximal antifungal therapy and surgical debridement. The mainstay of therapy includes liposomal amphotericin B and surgical debridement. Adjunctive azole antifungals (posaconazole and isavuconazole) are used as salvage therapy or as part of a combined antifungal regimen [4, 9]. The registries concluded that outcomes are improved when antifungal therapy and surgery are combined [13]. When possible, removal of predisposing risk factors and rapid correction of metabolic derangements are beneficial for prevention of disease progression [4].

## Conclusion

Though glucocorticoids are reported as a risk factor in COVID-19-associated mucormycosis [21], they were not a contributing factor in the progression of our patient’s infection since she did not receive steroid treatment for her COVID-19 infection. Our patient’s presentation and review of the literature suggests that COVID-19 infection could have potentiated her underlying risk factors (long-standing hyperglycemia, ketoacidosis) for development of this severe infection through immune dysregulation and possible precipitation of ketoacidosis [1]. Early recognition, initiation of therapy, and surgical debridement are paramount in preventing progression of disease and decreasing mortality [9, 22]. Given the rare incidence and often fulminant progression, a high index of suspicion is warranted for early diagnosis and treatment [1, 14, 22].

## Data Availability

Not applicable.

## Abbreviations

DKA: Diabetic ketoacidosis
COVID-19: Coronavirus disease 2019
SARS-CoV-2: Severe acute respiratory syndrome coronavirus 2
HTS: Hypertonic saline
PICU: Pediatric intensive care unit
CT: Computed tomography
MRI: Magnetic resonance imaging
LP: Lumbar puncture
CSF: Cerebrospinal fluid
ENT: Otorhinolaryngology
EKG: Electrocardiogram
ACE: Angiotensin-converting enzyme
CotH: Coat homolog
GRP78: Glucose-regulated protein-78
OE: Olfactory epithelium
